# Vascular thrombosis after single dose Ad26.COV2.S vaccine in healthcare workers in South Africa: open label, single arm, phase 3B study (Sisonke study)

**DOI:** 10.1136/bmjmed-2022-000302

**Published:** 2023-03-23

**Authors:** Barry Frank Jacobson, Elise Schapkaitz, Azwi Takalani, Pradeep Rowji, Vernon Johan Louw, Jessica Opie, Linda-Gail Bekker, Nigel Garrett, Ameena Goga, Tarylee Reddy, Nonhlanhla Yende-Zuma, Ian Sanne, Ishen Seocharan, Jonny Peter, Michelle Robinson, Shirley Collie, Amber Khan, Simbarashe Takuva, Glenda Gray

**Affiliations:** 1 Molecular Medicine and Haematology, Faculty of Health Sciences, University of the Witwatersrand, Johannesburg, South Africa; 2 Hutchinson Centre Research Institute of South Africa (HCRISA), Chris Hani Baragwanath Hospital, Johannesburg, South Africa; 3 The Southern African Society of Thrombosis and Haemostasis, Neurology Association of South Africa, Johannesburg, South Africa; 4 Division of Clinical Haematology, Department of Medicine, Faculty of Health Sciences, University of Cape Town and Groote Schuur Hospital, Cape Town, South Africa; 5 Division of Haematology, Department of Pathology, University of Cape Town and National Health Laboratory Service, Cape Town, South Africa; 6 Desmond Tutu HIV Centre, University of Cape Town, Cape Town, South Africa; 7 Centre for the AIDS Programme of Research in South Africa, Durban, South Africa; 8 Discipline of Public Health Medicine, School of Nursing and Public Health, University of KwaZulu-Natal, Durban, South Africa; 9 HIV and Other Infectious Diseases Research Unit, South African Medical Research Council, Cape Town, South Africa; 10 Paediatrics and Child Health, University of Pretoria, Pretoria, South Africa; 11 Biostatistics Research Unit, South African Medical Research Council, Durban, South Africa; 12 Nelson R Mandela School of Medicine, Centre for the AIDS Programme of Research in South Africa, University of KwaZulu Natal, Durban, South Africa; 13 Clinical HIV Research Unit, University of the Witwatersrand Faculty of Sciences, Johannesburg, South Africa; 14 Division of Allergy and Clinical Immunology, Faculty of Health Sciences, University of Cape Town, Cape Town, South Africa; 15 Allergy and Immunology Unit, University of Cape Town Lung Institute, Cape Town, South Africa; 16 Right To Care, Johannesburg, South Africa; 17 Discovery Health, Johannesburg, South Africa; 18 School of Health Systems and Public Health, University of Pretoria, Faculty of Health Sciences, Pretoria, South Africa; 19 Perinatal HIV Research Unit, University of the Witwatersrand, Faculty of Health Sciences, Johannesburg, South Africa

**Keywords:** Clinical trial, COVID-19, Hematologic diseases, Thromboembolism

## Abstract

**Objective:**

To assess the rates of vascular thrombotic adverse events in the first 35 days after one dose of the Ad26.COV2.S vaccine (Janssen/Johnson & Johnson) in healthcare workers in South Africa and to compare these rates with those observed in the general population.

**Design:**

Open label, single arm, phase 3B study.

**Setting:**

Sisonke study, South Africa, 17 February to 15 June 2021.

**Participants:**

The Sisonke cohort of 477 234 healthcare workers, aged ≥18 years, who received one dose of the Ad26.COV2.S vaccine.

**Main outcome measures:**

Observed rates of venous arterial thromboembolism and vaccine induced immune thrombocytopenia and thrombosis in individuals who were vaccinated, compared with expected rates, based on age and sex specific background rates from the Clinical Practice Research Datalink GOLD database (database of longitudinal routinely collected electronic health records from UK primary care practices using Vision general practice patient management software).

**Results:**

Most of the study participants were women (74.9%) and median age was 42 years (interquartile range 33-51). Twenty nine (30.6 per 100 000 person years, 95% confidence interval 20.5 to 44.0) vascular thrombotic events occurred at a median of 14 days (7-29) after vaccination. Of these 29 participants, 93.1% were women, median age 46 (37-55) years, and 51.7% had comorbidities. The observed to expected ratios for cerebral venous sinus thrombosis with thrombocytopenia and pulmonary embolism with thrombocytopenia were 10.6 (95% confidence interval 0.3 to 58.8) and 1.2 (0.1 to 6.5), respectively. Because of the small number of adverse events and wide confidence intervals, no conclusions were drawn between these estimates and the expected incidence rates in the population.

**Conclusions:**

Vaccine induced immune thrombocytopenia and thrombosis after one dose of the Ad26.COV2.S vaccine was found in only a few patients in this South African population of healthcare workers. These findings are reassuring if considered in terms of the beneficial effects of vaccination against covid-19 disease. These data support the continued use of this vaccine, but surveillance is recommended to identify other incidences of venous and arterial thromboembolism and to improve confidence in the data estimates.

**Trial registration:**

ClinicalTrials.gov NCT04838795.

**WHAT IS ALREADY KNOWN ON THIS TOPIC**

- In adults, the evidence suggests that the Ad26.COV2.S vaccine (Janssen/Johnson & Johnson) increases the risk of vaccine induced immune thrombocytopenia and thrombosis
- Data on the safety of vaccination with Ad26.COV2.S are limited and a direct effect on the development of vaccine induced immune thrombocytopenia and thrombosis has not been established in African populations
- Cohort single and multicentre studies, and reviews of vaccine induced immune thrombocytopenia and thrombosis after vaccination with Ad26.COV2.S prompted a cautionary recommendation by the US Centers for Disease Control and Prevention advisory committee that was endorsed by the Food and Drug Administration

**WHAT THIS STUDY ADDS**

- Vaccine induced immune thrombocytopenia and thrombosis was found in only a few particpants among South African healthcare workers
- The risk of total venous or arterial thromboembolism events was not increased in this large population of healthcare workers who were vaccinated with the Ad26.COV2.S vaccine

**HOW THIS STUDY MIGHT AFFECT RESEARCH, PRACTICE, OR POLICY**

- Increased risk of vaccine induced immune thrombocytopenia and thrombosis with the Ad26.COV2.S vaccine in adults emphasises the importance of diagnosis and management in accordance with recommendations
- The risk-to-benefit ratio of vaccination with the Ad26.COV2.S vaccine on a population level is encouraging
- The Ad26.COV2.S vaccine represents a practical solution for Africa and countries with limited resources, owing to the less stringent storage requirements and single dose scheduling

## Introduction

The efficacy of vaccines against SARS-CoV-2 has been reported to be 53-97% in patients with symptoms of infection.^1–6^ Of these, the adenovirus vector vaccines are the preferred vaccines in countries with limited resources because of their accessibility and less stringent storage requirements. Two of these include the chimpanzee adenoviral vector, ChAdOx1 nCoV-19 vaccine (Oxford-AstraZeneca) and the adenoviral vector type 26, Ad26.COV2.S vaccine (Janssen/Johnson & Johnson). The risk of adverse events after these vaccines has been assessed in clinical trials and by public health surveillance systems. Reports of serious adverse events have been rare. Nonetheless, some instances of venous thromboembolism and arterial thrombosis after adenoviral vector vaccines have been reported, raising concerns about the potential risks of thromboembolic complications.^7–10^

Moderate-to-severe thrombocytopenia and thrombosis at unusual sites have been reported in rare instances after vaccination with the ChAdOx1 nCoV-19 and Ad26.COV2.S vaccines.^11 12^ This finding prompted a recommendation by several regulatory authorities for a temporary suspension of vaccinations in April 2021.^13^ The term vaccine induced immune thrombocytopenia and thrombosis was created for this syndrome, with a proposed mechanism similar to that of heparin induced thrombocytopenia with thrombosis.^14 15^ Also, the term thrombosis with thrombocytopenia syndrome has been defined by the Brighton Collaboration.^16^ Thrombosis with thrombocytopenia syndrome can be attributed to several causes and the term is recommended for use in epidemiological studies after vaccination.

Definite or probable vaccine induced immune thrombocytopenia and thrombosis was reported in 220 patients presenting to hospitals in the UK after 24 million first doses of the ChAdOx1 nCoV-19 vaccine.^17^ Median age of these patients was 48 years (range 18-79) and 55% were women. Of these, 209 (95%) presented with thrombocytopenia (median 47×10^9^/L, range 28-76) and 102 (46%) with cerebral venous sinus thrombosis. No other risk factors for thrombosis were identified and the overall mortality rate was 22%. Up to 8 July 2021, another 38 patients with definite vaccine induced immune thrombocytopenia and thrombosis after 12.5 million first doses of Ad26.COV2.S vaccine were reported to the US Centers for Disease Control and Prevention advisory committee on immunisation practices.^18^ These patients had a similar presentation to those described after vaccination with the ChAdOx1 nCoV-19 vaccine, suggesting an effect of adenoviral vector-type vaccines against SARS-CoV-2. Thrombosis at unusual sites and thrombocytopenia have also been reported for mRNA based vaccines, although to a lesser extent.^10 19^ Given the severity of vaccine induced immune thrombocytopenia and thrombosis and the ongoing covid-19 pandemic, close monitoring and reporting of serious adverse events is essential.

Between 17 February and 17 May 2021, the Ad26.COV2.S vaccine was given to 477 234 individuals in the Sisonke study, a multicentre, open label, single arm, phase 3B implementation study in healthcare workers in South Africa. Preliminary data on vascular thrombotic events after vaccination of the first 288 368 healthcare workers have been reported.^20^ The rate of venous and arterial thromboembolic events were 1.7 per 100 000 participants. These events occurred in individuals with known risk factors for thromboembolism. Here, we describe the occurrence of vascular thrombotic events after vaccination of 477 234 healthcare workers in the Sisonke study, including two patients with vaccine induced immune thrombocytopenia and thrombosis.

## Methods

### Study participants and procedures

The Sisonke study has been previously described.^21^ After the temporary suspension of vaccinations in South Africa on 13 April 2021, vaccinations resumed on 28 April 2021, with further safeguards. These safeguards included: changes to the eligibility criteria in the Sisonke protocol; more information for participants about the signs and symptoms of thrombosis; amendments to patient information sheets and informed consent forms; and modified standard operating procedures with training to support appropriate screening as well as management of participants at high risk of thrombosis. Also, a short message surveillance service was implemented with specific increased awareness of the risk of thrombosis.

Vaccinations were conducted in collaboration with the South African Department of Health in public or private vaccination centres nationwide. Delivery of the vaccine was managed by appropriately trained personnel linked to the ENSEMBLE (A Study of Ad26.COV2.S for the Prevention of SARS-CoV-2-Mediated COVID-19 in Adult Participants) trial research sites (ClinicalTrials.gov NCT04505722). All healthcare workers, registered with the Electronic Vaccination Data System, the South African national vaccination registry, and who provided consent for the study electronically were eligible for enrolment. The study excluded women who were breastfeeding (after 28 April 2021) and pregnant women because of lack of sufficient safety data.

Participants received one intramuscular injection of Ad26.COV2.S at a dose of 5×10^10^ virus particles, and were observed for adverse events for 15 minutes after vaccination, or for 30 minutes if they had a previous history of allergic reactions to vaccinations. The Sisonke study was a single dose study. A second dose study, Sisonke, 2 was performed after findings from international studies showed the protective effect of a second dose.^22^

### Safety monitoring

Adverse events in the Sisonke study were reported from a hybrid surveillance system that combined passive and active reporting of adverse event outcomes. Full details of the Sisonke pharmacovigilance and safety reporting processes are detailed elsewhere.^21^ Adverse event reports were reviewed and processed daily by the safety team, and validated and updated by telephonic interviews with participants, healthcare providers, and from accessible medical records. A safety review team of public health experts and specialists in neurology and haematology met weekly to review the cumulative safety data and case summaries. An independent safety monitoring committee provided safety oversight. In this analysis, we evaluated all data up to 15 June 2021 (35 days after the last vaccination). Surveillance for adverse event is ongoing and will continue for up to two years after vaccination.

### Case definitions

Safety events of concern included adverse events of special interest: vascular thrombosis after vaccination, based on the Brighton Collaboration list, and serious adverse events after vaccination that met the criteria of the International Conference on Harmonisation.^23^ Vascular thrombosis included one or more episodes of venous, arterial, or small vessel thrombosis. Venous thromboembolism events were radiologically confirmed by cerebral magnetic resonance venography and compression ultrasound of the limbs, with or without computed tomography angiography of the chest and abdomen. Arterial thrombosis events included acute coronary syndrome diagnosed by electrocardiogram and cardiac biomarkers, and ischaemic stroke diagnosed by neurological examination in combination with computed tomography or magnetic resonance imaging. Small vessel thrombosis was diagnosed by histopathology in any tissue or organ.

Definite vaccine induced immune thrombocytopenia and thrombosis was defined as: onset of symptoms 5-30 days after vaccination, presence of thrombosis, platelet count <150×10^9^/L or decrease of 50% from baseline, and presence of antibodies to platelet factor 4.^24 25^ The diagnosis was further supported by plasma concentrations of d-dimers >0.25 mg/L and fibrinogen <2 g/L. If two of the four criteria were met, participants were defined as having possible vaccine induced immune thrombocytopenia and thrombosis (online supplemental tables 1S and 2S).^17^

10.1136/bmjmed-2022-000302.supp1 Supplementary data

### Blood sampling and laboratory methods

Testing for the SARS-CoV-2 virus was performed in all participants with a real time polymerase chain reaction test of the upper respiratory tract (nasopharyngeal and oropharyngeal swab). As far as possible, serological testing was performed. Antibodies to platelet factor 4 were detected by commercial platelet factor 4/heparin enzyme linked immunoassays (Zymutest HIA IgG; Hyphen BioMed, Neuville-sur-Oise, France).^25^ The positive threshold was based on the manufacturer’s optical density threshold (reference range <0.30 negative, 0.3-0.5 weak positive, and >0.5 strong positive). Positive results were confirmed by a functional assay^26 27^ (platelet aggregation profiler, PAP-8E, reference range <25%; Bio/Data Corporation, Horsham, PA) at a centralised referral laboratory.

### Data analysis

For all data transfer during the study, industry standard multilayer encryption platforms were used. The Electronic Vaccination Data System used a beneficiary identifier, a unique 36 character machine generated identifier within the system. This identifier was used for data linkages, removing any direct identifiers.

Statistical analyses were conducted with Stata, version 14. For descriptive statistics, frequencies and percentages were used for categorical variables and median (interquartile range) for continuous variables. Occurrence of venous and arterial thromboembolic events were described by baseline characteristics of participants. The observed number of reported incidences was compared with the expected number based on background incidence rates, and the observed to expected ratio (with 95% confidence interval) was calculated. The expected number of events was estimated based on background incidence rates of vascular thrombotic events in the Clinical Practice Research Datalink GOLD database (database of longitudinal routinely collected electronic health records from UK primary care practices using Vision general practice patient management software).^28 29^ This database contains data from 3 913 071 participants with a median age of 41 years (interquartile range 22-59), contributed by general practitioners from the UK between 1 January 2017 and 31 December 2019. These background incidence rates were applied because no data were available locally. The Clinical Practice Research Datalink GOLD database was selected because it includes thromboses of special interest for covid-19 vaccinations, with rates grouped by sex and age.^30^ Person time was accrued from the date of vaccination until death or closure of the dataset on 15 June 2021 (35 days after the last vaccination). The incidence rates per 100 000 person years were calculated with a Poisson model, with person years as an offset.

### Patient and public involvement

No patients were involved in the design, execution, or interpretation of this study. Owing to the urgency and sensitivity of the study question, as well as data privacy constraints, involving members of the public in the study was not possible. The results of the study have been disseminated to research participants and public communities.electronically as well as via the press, parliamentary committees, and annual reports.

## Results

### Participant characteristics

**Figure 1:**
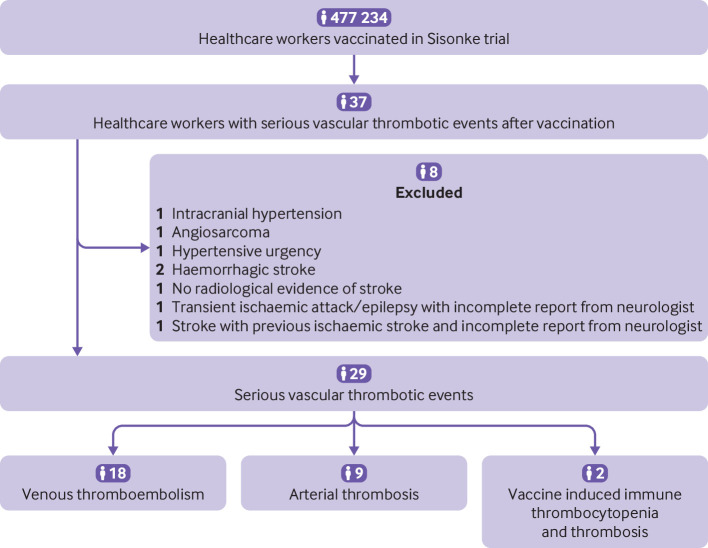
Flowchart of study participants

The Sisonke cohort included 119 753 (25.1%) male participants and 357 481 (74.9%) female participants, with a median age of 42 (interquartile range 33-51) years. Data for sex were taken from information in the Sisonke study rather than from patient reported gender. The higher proportion of women was because the nursing profession in South Africa has disproportionately more women than men. Vascular thrombotic events were reported for 37 participants. Eight participants were excluded (figure 1) and 29 (30.6 per 100 000 person years, 95% confidence interval 20.5 to 44.0) vascular thrombotic events were included. Table 1 shows the baseline characteristics of the 29 participants. Most participants were women (n=27, 93.1%) and median age was 46 years (interquartile range 37-55). Median time from vaccination to onset of thrombosis was 14 days (7-29). Baseline characteristics of participants who presented with venous thromboembolism, arterial thrombosis, or vaccine induced immune thrombocytopenia and thrombosis were not substantially different.

**Table 1:** Baseline characteristics of participants with venous thromboembolism, arterial thrombosis, and vaccine induced immune thrombocytopenia and thrombosis in the Sisonke study

Characteristics	Sisonke study	All thrombotic events	Venous thromboembolism	Arterial thrombosis	Vaccine induced immune thrombocytopenia and thrombosis
Total No of participants	477 234	29 (100)	18 (62.1)	9 (31.0)	2 (6.9)
Sex:					
Men	119 753 (25.1)	2 (6.9)	1 (5.6)	1 (11.1)	0 (0)
Women	357 481 (74.9)	27 (93.1)	17 (94.4)	8 (88.9)	2 (100.0)
Age group (years):					
18-39	209 411 (43.9)	10 (34.5)	7 (38.9)	2 (22.2)	1 (50.0)
40-49	136 967 (28.7)	10 (34.5)	6 (33.3)	3 (22.2)	1 (50.0)
50-59	96 235 (20.2)	3 (10.3)	0 (0.0)	3 (33.3)	0 (0.0)
≥60	23 389 (7.2)	6 (20.7)	5 (27.8%)	1 (11.1%)	0 (0.0)
Median (IQR) age (years)	42 (33-51)	46 (37-55)	44 (34-60)	46 (43-55)	39 (28-49)
Median (IQR) time to onset of thrombosis (days)	–	14 (7-29)	16 (7-23)	10 (5-38)	19 (7-30)
Comorbidities:					
Cancer	1 364 (0.3)	0 (0)	0 (0)	0 (0)	0 (0)
Chronic lung disease	1 733 (0.4)	0 (0)	1 (5.6)	0 (0)	0 (0)
Diabetes	28 063 (5.9)	3 (10.3)	1 (5.6)	0 (0)	0 (0)
Heart disease	3 430 (0.7)	2 (6.9)	0 (0)	1 (8.3)	0 (0)
HIV	39 386 (8.3)	2 (6.9)	1 (5.6)	1 (8.3)	0 (0.)
Hypertension	74 381 (15.6)	8 (27.6)	3 (16.7)	3 (33.3)	0 (0)
Tuberculosis	478 (0.1)	0 (0)	0 (0)	0 (0)	0 (0)

Data are number (%) unless stated otherwise.

IQR=interquartile range.

### Description of vascular thrombotic events

**Figure 2:**
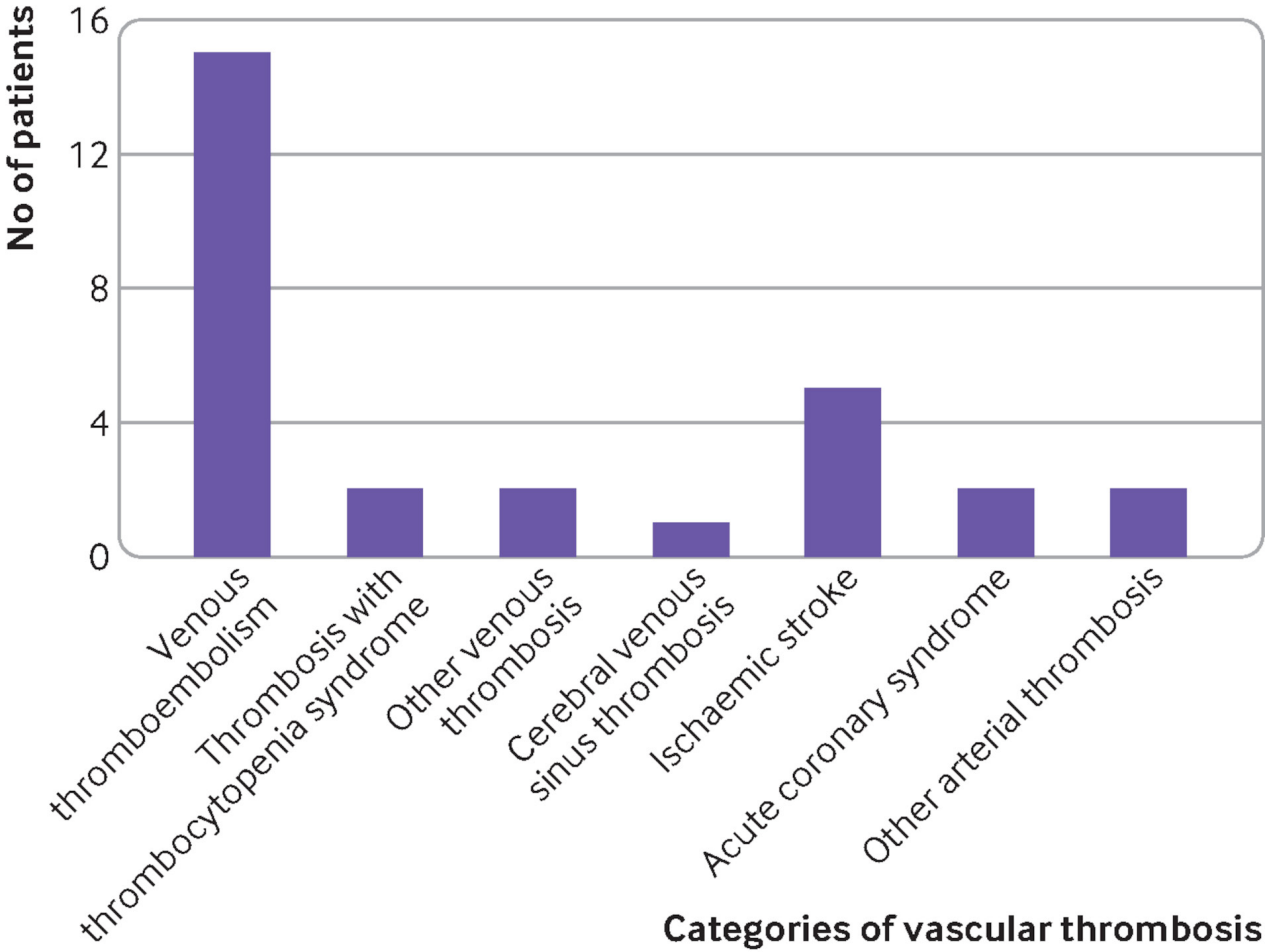
Categories of vascular thrombosis. Other arterial thrombosis included cephalic vein thrombosis (n=1) and retinal vein thrombosis (n=1)

Eighteen (62.1%) venous thromboembolism events were observed. Of these, pulmonary embolism and deep vein thrombosis were the most common venous events, affecting 15 (83.3%) of the 18 participants (figure 2). Other venous sites were uncommon presentations: cephalic vein thrombosis (n=1, 5.6%) and retinal vein thrombosis (n=1, 5.6%). One (5.6%) cerebral venous sinus thrombosis event was reported in a woman (aged 30-39 years). The woman presented with a persistent headache followed by a seizure 29 days after vaccination, associated with a normal platelet count and negative antiplatelet factor 4 antibodies. When the analysis was grouped by age and sex, the observed to expected ratio for cerebral venous sinus thrombosis was 2.8 (95% confidence interval 0.1 to 15.4) for women aged 30-39 years.

Of the nine (31.0%) arterial thrombotic events, five (55.6%) were ischaemic strokes, two (22.2%) were acute coronary syndrome, and one (11.1%) was an ischaemic limb associated with positive antiphospholipid antibodies. Also, one patient (11.1%) had leucocytoclastic vasculitis, diagnosed by histopathological examination of a biopsy sample. We found no evidence of a clear association between previous vaccination with the Ad26.COV2.S vaccine and arterial thrombosis after vaccination. When the analysis was grouped by age and sex, the observed to expected ratio for ischaemic strokes was 2.5 (95% confidence interval 0.3 to 9.0) for women aged 30-39 years and 1.2 (0.1 to 4.2) for women aged 40-49 years.

Of the 29 participants who presented with vascular thrombotic events, two (6.9%) fulfilled the diagnostic criteria for definite or possible vaccine induced immune thrombocytopenia and thrombosis; the first had a pulmonary embolus with thrombocytopenia and the second a cerebral sinus venous thrombosis with thrombocytopenia. These results corresponded to an observed to expected ratio of 1.2 (95% confidence interval 0.1 to 6.5) and 10.6 (0.3 to 58.8), respectively. The first participant was a woman (aged 40-49 years) who presented with a saddle pulmonary embolism, platelet count of 34×10^9^/L, positive antiplatelet factor 4 antibodies of 0.86, confirmed by a functional heparin induced thrombocytopenia with thrombosis assay (>80%), and plasma concentrations of d-dimers >10.0 mg/L, nine days after vaccination. The second participant was a woman (aged 20-29 years) who was admitted to hospital unconscious after a severe headache, restlessness, and confusion, one month after vaccination. A computed tomography venogram was consistent with cerebral venous sinus thrombosis. The antiplatelet factor 4 antibody assay was negative, and she had marginal thrombocytopenia (146×10^9^/L). Both participants recovered.

At the time of the data analysis, two deaths (6.9%) related to vascular thrombosis were reported. The first participant was a woman (aged 60-69 years) with a previous history of venous thromboembolism, who collapsed 23 days after vaccination. A pulmonary embolism was diagnosed on postmortem examination. The second participant was a woman (aged 30-39 years) who presented with a pulmonary embolism, 41 days after vaccination. She died after admission to hospital, 42 days after vaccination.

### Risk factors for vascular thrombotic events

Of the 29 vascular events, 15 (51.7%) occurred in participants with one or more comorbidities (table 2), and four (13.8%) participants had a history of thrombosis.

**Table 2:** Observed versus expected vascular thrombotic events after vaccination with the Ad26.COV2.S vaccine in the Sisonke study, by sex and age

	Observed count	Person years	Observed incidence rate per 100 000 person years (95% CI)	Expected incidence rate per 100 000 person years (95% CI)	Expected count	Observed to expected ratio (95% CI)
**Venous thromboembolism**						
Total No of pulmonary embolism events	11	94 743.07	11.61 (6.43 to 20.97)	74.0 (72.3 to 75.7)	70.11	0.16 (0.08 to 0.28)
Men:						
Aged 60-69 years	1	1677.95	59.60 (8.39 to 423.08)	151.3 (141.1 to 162.1)	2.54	0.39 (0.01 to 2.19)
Women:						
Aged 30-39 years	3	20 135.48	14.90 (4.81 to 46.20)	40.1 (35.4 to 45.2)	8.07	0.37 (0.08 to 1.09)
Aged 40-49 years	4	21 204.73	18.86 (7.08 to 50.26)	50.1 (44.9 to 55.8)	10.62	0.38 (0.10 to 0.96)
Aged 60-69 years	3	4137.99	72.50 (23.38 to 224.79)	134.9 (125.4 to 145.0)	5.58	0.54 (0.11 to 1.57)
Total No of deep vein thrombosis events	4	94 743.07	4.22 (1.58 to 11.25)	93.9 (92.0 to 95.9)	88.96	0.04 (0.01 to 0.12)
Women:						
Aged 20-29 years	1	9989.95	10.01 (1.41 to 71.06)	58.5 (52.4 to 65.2)	5.84	0.17 (0 to 0.95)
Aged 30-39 years	2	20 135.48	9.93 (2.48 to 39.72)	71.8 (65.5 to 78.6)	14.46	0.14 (0.02 to 0.5)
Aged 40-49 years	1	21 204.73	4.72 (0.66 to 33.48)	80.4 (73.7 to 87.5)	17.05	0.06 (0 to 0.33)
Total No of cerebral venous sinus thrombosis events	1	94 743.07	1.06 (0.15 to 7.49)	1.2 (1.0 to 1.5)	1.14	0.88 (0.02 to 4.9)
Women:						
Aged 30-39 years	1	20 135.48	4.97 (0.70 to 35.26)	1.8 (0.9 to 3.2)	0.36	2.76 (0.07 to 15.37)
**Arterial thrombosis**						
Total No of ischaemic stroke events	5	94 743.07	5.28 (2.20 to 12.68)	29.6 (28.5 to 30.7)	28.04	0.18 (0.06 to 0.42)
Women:						
Aged 30-39 years	2	20 135.48	9.93 (2.48 to 39.72)	4.0 (2.6 to 5.8)	0.81	2.48 (0.30 to 8.97)
Aged 40-49 years	2	21 204.73	9.43 (2.36 to 37.71)	8.2 (6.2 to 10.7)	1.74	1.15 (0.14 to 4.16)
Aged 50-59 years	1	15 054.54	6.64 (0.94 to 47.16)	17.3 (14.4 to 20.7)	2.6	0.38 (0.01 to 2.14)
Total No of acute coronary syndrome events	2	94 743.07	2.11 (0.53 to 8.44)	138.8 (136.5 to 141.2)	131.5	0.02 (0 to 0.05)
Men:						
Aged 60-69 years	1	1677.95	59.60 (8.39 to 423.08)	437.2 (419.7 to 455.4)	7.34	0.14 (0 to 0.76)
Women:						
Aged 40-49 years	1	21 204.73	4.72 (0.66 to 33.48)	33.9 (29.7 to 38.7)	7.19	0.14 (0 to 0.78)
**Vaccine induced immune thrombocytopenia and thrombosis**						
Total No of pulmonary embolism and thrombocytopenia events	1	94 743.07	1.06 (0.15 to 7.49)	0.9 (0.7 to 1.1)	0.85	1.17 (0.03 to 6.53)
Women:						
Aged 40-49 years	1	21 204.73	4.72 (0.66 to 33.48)	0.3 (0.1 to 0.8)	0.06	15.72 (0.4 to 87.58)
Total No of cerebral sinus venous thrombosis and thrombocytopenia events	1	94 743.07	1.06 (0.15 to 7.49)	0.1 (0.1 to 0.2)	0.09	10.55 (0.27 to 58.81)
Women:						
Aged 20-29 years	1	9989.98	10.01 (1.41 to 71.06)	–	–	–

CI=confidence interval.

## Discussion

### Principal findings

Safety concerns about vascular thrombotic events have been raised after vaccination with adenoviral vector vaccines against SARS-CoV-2 infection. Venous thromboembolism, arterial thrombosis, and vaccine induced immune thrombocytopenia and thrombosis events were observed after the first dose of the Ad26.COV2.S vaccine. This finding prompted a cautionary recommendation by the US Centers for Disease Control and Prevention advisory committee which was endorsed by the Food and Drug Administration. This South African population based study of almost half a million healthcare workers, aged ≥18 years, who received the Ad26.COV2.S vaccine, provides further real world evidence that the risk of vascular thrombotic events seems to be low, supporting its continued use in this setting.

### Comparison with other studies

Venous thromboembolism is among the most common causes of morbidity and mortality in Africa.^31^ When considering the effect of vaccination on public health, the background incidence rate of thrombosis in the general population, as well as according to age and sex, should be taken into account. Consistent with other population based studies, the most common thrombotic event in this study population was venous thromboembolism.^7–9^ The rates for total venous thromboembolism events were below the expected background incidence rates of the Clinical Practice Research Datalink GOLD database. This finding is in contrast with the substantially increased rates of venous thromboembolism secondary to covid-19 disease.^32–34^ In particular, earlier strains of the SARS-CoV-2 virus were associated with an increased rate of pulmonary embolism (>30%) in patients admitted to hospital.^35–37^ Nonetheless, we found an increase in the incidence of definite or possible vaccine induced immune thrombocytopenia and thrombosis. Vaccine induced immune thrombocytopenia and thrombosis has consistently been reported as a rare event in the general population.^11 12^ These two (6.9%) patients (pulmonary embolus with thrombocytopenia and cerebral sinus venous thrombosis with thrombocytopenia) were diagnosed and managed with the support of the protocol safety review team, and both participants recovered. Timely identification of these events will ensure the successful management of vaccine induced immune thrombocytopenia and thrombosis according to published recommendations.^25^

Furthermore, we found no increase in the rates of arterial thromboembolism. Our subanalysis according to age and sex, in women aged 30-39 years, showed three ischaemic strokes in the vaccine cohort compared with one expected event. This finding should be interpreted in terms of recently published data which reported a 2-3-fold higher incidence of stroke in Africa than in Europe.^38^ Also, more than half of the vascular thrombotic events in our study occurred in participants with one or more cardiovascular risk factors. Moreover, according to a recent meta-analysis, patients with severe covid-19 disease have a fivefold increased risk of stroke.^39^ Specific immune mediated and prothrombotic mechanisms contribute to the increased risk of vascular thrombotic events, providing another clear reason for vaccination.

### Strengths and limitations of this study

The findings of the Sisonke study have important regulatory implications. The Ad26.COV2.S vaccine showed >60% protection against severe covid-19 disease and >80% protection against death related to SARS-CoV-2 infection.^40^ Therefore, the risk-to-benefit ratio of vaccination at the population level is encouraging. From a public health perspective, the Ad26.COV2.S vaccine represents a practical solution for Africa, with limited resources,^22^ owing to the less stringent storage requirements and single dose scheduling associated with reasonable efficacy.^40^

Our study had several limitations. Firstly, the Sisonke study is mainly a passive surveillance system, relying on self-reporting, and therefore adverse events after vaccination with the Ad26.COV2.S vaccine might have been under-reported. Because the study cohort were heathcare workers, however, adverse events would likely have been reported, with the the potential for overreporting of adverse events. Also, the temporary suspension in adenovirus based vaccinations in April 2021 could have increased participants’ levels of clinical awareness and self-reporting of adverse events. In our study, comparison of adverse events with mRNA based vaccines was not possible because Ad26.COV2.S was the only available vaccine at the time of this study. In the study of Cari et al,^6^ to reduce under-reporting and overreporting biases, adverse events with a mRNA based covid-19 vaccine were compared with adenovirus based vaccines. All adverse events assessed in this study were verified by our study investigators.

Secondly, the observed to expected analysis should be considered in terms of the non-South African reference data. The background incidence rates of the GOLD database were selected because they included thromboses of special interest for covid-19 vaccinations, with rates grouped by sex and age. Subsequently, Discovery Health conducted a study of South African medical insurance claims for adverse events related to the Ad26.COV2.S vaccine between February 2021 and September 2021. This study compared adverse events (venous thromboembolic events, stroke, and myocardial infarction) in 103 315 recipients of a vaccine and matched individuals who were not vaccinated (online supplemental table 4) but no signifcant differences were found.

Thirdly, owing to the small number of adverse events, comparisons with the incidence in the expected population were associated with wide 95% confidence intervals and therefore no conclusions could be drawn from the data. Ongoing surveillance is needed to identify further venous and arterial thromboembolism events and to improve the accuracy of the data estimates. Fourthly, the Sisonke study did not report participants’ race and therefore adverse events could not be grouped by race. Lastly, risk factors for venous thromboembolism, such as history of thrombosis, inherited thrombophilia, and use of oral contraceptives, were not available for all participants.

### Conclusions

In our pharmacovigilance study of single dose Ad26.COV2.S vaccine, we found two patients with vaccine induced immune thrombocytopenia and thrombosis among almost half a million recipients. The risks of total venous or arterial thromboembolism events were also low. The incidence of cerebral and splanchnic venous thrombosis with or without thrombocytopenia seemed to be lower after vaccination with the Ad26.COV2.S vaccine than the ChAdOx1 nCoV-19 vaccine, as reported by the EudraVigilance European database.^19^ These risks, when interpreted with data on the effectiveness of the Ad26-CoV2.S vaccine against severe disease and death, emphasise the benefit versus risk of the Ad26.CoV2.S vaccine at the population level.

## Data Availability

All data relevant to the study are included in the article or uploaded as supplementary information.
