# Supplementary material for: Vascular thrombosis after single dose Ad26.COV2.S vaccine in healthcare workers in South Africa: open label, single arm, phase 3B study (Sisonke study)
Source: BMJ Med. 2023 Mar 23;2(1):e000302. doi: 10.1136/bmjmed-2022-000302 (PMC10083528; doi:10.1136/bmjmed-2022-000302)
Supplement: Supplementary data [file bmjmed-2022-000302supp001.pdf]

Table 1S | Definitions of Vaccine-induced Immune Thrombocytopenia and Thrombosis (VITT) according to Expert Haematology Panel [17]

Definite VITT	All five criteria:<br>1. Onset of symptoms 5–30 days after vaccination against SARS-CoV-2 (or ≤42 days in patients with isolated deep-vein thrombosis or pulmonary embolism)<br>2. Presence of thrombosis<br>3. Thrombocytopenia (platelet count <150 x 10^9/L)<br>4. D-dimer> 4000 FEU<br>5. Positive anti-PF4 antibodies on ELISA
Probable VITT	D-dimer level >4000 FEU but one criterion not met (timing, thrombosis, thrombocytopenia, or anti-PF4 antibodies) or d-dimer level unknown or 2000–4000 FEU and all other criteria met
Possible VITT	D-dimer level unknown or 2000–4000 FEU with one other criterion not met, or two other criteria not met (timing, thrombosis, thrombocytopenia, or anti-PF4 antibodies)
Unlikely VITT	Platelet count <150 x 10^9/L and d-dimer level <2000 FEU, or thrombosis with platelet count >150 x 10^9/L and d-dimer level <2000 FEU, regardless of anti-PF4 antibody result, and alternative diagnosis more likely

Key: ELISA, enzyme-linked immunosorbent assay; FEU, fibrinogen-equivalent unit; PF4. platelet factor 4 and SARS-CoV-2, severe acute respiratory syndrome coronavirus 2.

Table 2S | Brighton Collaboration interim case definition of Thrombosis with Thrombocytopenia

Confirmed TTS	Presence of major criteria:<br>1. presence of new onset severe thrombocytopenia defined as a platelet count <50 x 10^9/L, and<br>2. new thrombosis at uncommon site: cerebral veins, splanchnic veins or multiple organ<br>3. Positive anti-platelet factor 4 antibodies
Probable TTS	Presence of major and minor criteria:<br>1. presence of new onset severe thrombocytopenia defined as a platelet count <50 x 10^9/L, and<br>2. new thrombosis at common site: pulmonary arteries/veins, limb veins, coronary arteries, cerebral arteries or other arteries/vein<br>OR 1. presence of new onset mild to moderate thrombocytopenia defined as a platelet count <150 x10^9/L, and<br>2. new thrombosis at uncommon site.
Possible TTS	Presence of minor criteria:<br>1. presence of new onset mild to moderate thrombocytopenia defined as a platelet count <150 x 109/L, and<br>2. new thrombosis at common site.<br>or<br>1. presence of new onset thrombocytopenia defined as a platelet count <150 or >50 x 109/L, or >50% decrease from baseline platelet count and<br>2. suggestive thrombosis by supporting imaging or laboratory findings or by clinical syndromes in any location<br>3. D-dimer > 4000 FEU

Supplementary material

Clinical twin study

Data & Methodology

We assessed the risk of 179 conditions post vaccination with AD26.COV2.s amongst Discovery Health clients from 17 February 2021 to 15 September 2021, 18 years and older that had at least 6 months of exposure on Discovery Health. The list of conditions includes 25 conditions analysed in a real world safety study of BNT162b2 vaccination in Israel (Noam Barda, 2021), and a further 154 conditions which are considered immune mediated conditions (Table 4a). Real world safety studies amongst large populations are more likely to provide sufficient volumes to power risk ratio estimates of rare adverse events amongst the vaccinated population compared to randomized control studies which typically have lower volumes. The safety and immunogenicity study of the AD26.COV2.s vaccine was based on 43, 783 participants, split equally between those vaccinated with and those vaccinated with placebo (Sadoff, 2021). Comparison against unvaccinated controls provides greater certainty that identified adverse events are vaccine induced as it provides a risk relative to the population's baseline risk over a consistent timeframe. Registries of voluntary vaccine safety adverse events such as VAERS and the reporting system managed by the South African Health Products Regulatory Agency, unfortunately lack a relevant temporal comparison of underlying adverse events in the unvaccinated general population. This study compares adverse events amongst the vaccinated population against 'clinical twins' who did not receive vaccination The 'clinical twin' approach was based on the following matching criteria:

- **Age**: clinical twins were matched within one year of age of each other
- **Member's sex**: Male (M) or Female (F)
- **Location**: Hospital service area in South Africa
- **Number of chronic conditions**: 0, 1, 2, 3, 4 or more
- **Number of prior flu vaccinations**: 0, 1, 2, 3, 4, 5 or more. People who had a flu vaccination in the years leading up to and during the COVID-19 pandemic were likely to be more aware of promoting their general health and wellbeing and adhere to preventive measures.
- **One of five medical scheme product options**: Looking at out-of-hospital benefits we divided the 24 plans available through both Discovery Health Medical Scheme and 18 of the Discovery Health administered schemes, into 5 groups.

We assessed the rate of 179 adverse events in the vaccinated population, from the date of vaccination until 42 days after receipt of vaccination to matched unvaccinated 'clinical twin' controls. For each condition assessed, clients with a history of the condition were excluded from the analysis i.e. both the vaccinated and matched unvaccinated controls had no prior history of the condition, resulting in the new onset of the condition of interest being analysed after vaccination, relative to the matched baseline population. Individuals with a history of any prior COVID19 vaccination were excluded.

To obtain confidence intervals around risk ratio estimates and the number of adverse events in the study and comparator populations, we bootstrapped amongst our vaccinated population with replacement 500 times. Within each bootstrap vaccinated individuals were matched to a clinical twin, randomly. Once a clinical twin was selected, it was not eligible for selection again within a particular bootstrap. The 95% confidence interval was obtained by the following formula for each of the respective measures analysed (i.e. the risk ratio and number adverse events) : $95\%CI = \mu \pm 1.96\sigma$, where μ is the mean and σ is the standard deviation across bootstraps.

Results

103,315 vaccinated individuals were included in the study. There was a higher proportion of females included (67.6%), as the majority of vaccine recipients were healthcare workers vaccinated under the Sisonke clinical trial, and the nursing profession in South Africa is disproportionately weighted towards females (Table 4b). None of the 179 conditions evaluated amongst the vaccinated population had a statistically significant higher incidence risk ratio relative to unvaccinated controls (Table 4c).

Table 4a: List of analysed conditions

Pulmonary embolism	Evans syndrome	Sympathetic ophthalmia (SO)	Graves disease	Mucha-Habermann disease	Interstitial cystitis (IC)
Kawasaki disease	Immune thrombocytopenic purpura (ITP)	Optic neuritis	Ocular cicatricial pemphigoid	Pernicious anemia (PA)	Autoimmune retinopathy
Acute kidney injury	Thrombocytopenic purpura (TTP)	Leukocytoclastic vasculitis	Takayasus arteritis	Antiphospholipid syndrome	Sperm & testicular autoimmunity
Seizure	Herpes zoster infection	Hypersensitivity pneumonitis	Paraneoplastic cerebellar degeneration (PCD)	Neuromyelitis optica	Herpes simplex infection
Arrhythmia	PANDAS	Lichen sclerosus	Bells palsy	Myelin Oligodendrocyte Glycoprotein Antibody Disorder	Fibromyalgia
Juvenile diabetes (Type 1 diabetes)	Coxsackie myocarditis	Myasthenia gravis	Devics disease (neuromyelitis optica)	Anti-GBM/Anti-TBM nephritis	Autoimmune oophoritis
Type 1 diabetes	Chronic inflammatory demyelinating polyneuropathy (CIDP)	Achalasia	Lambert-Eaton syndrome	POEMS syndrome	Ligneous conjunctivitis
Anemia	Reactive Arthritis	Mixed connective tissue disease (MCTD)	Dresslers syndrome	Chagas disease	Rheumatoid arthritis
Syncope	Henoch-Schonlein purpura (HSP)	Undifferentiated connective tissue disease (UCTD)	Hypogammalglobulinemia	Lyme disease chronic	Progesterone dermatitis
Glomerulonephritis	Lupus	Erythema nodosum	Polyglandular syndromes type I, II, III	Adult Still's disease	Arthritis or arthropathy
Cerebrovascular accident	Polymyositis	Progressive systemic sclerosis	Churg-Strauss Syndrome (CSS) or Eosinophilic Granulomatosis (EGPA)	Polyarteritis nodosa	Autoimmune orchitis
Deep-vein thrombosis	Subacute bacterial endocarditis (SBE)	Ankylosing spondylitis	Granulomatosis with Polyangiitis	Lichen planus	Hyperthyroidism
Pericarditis	Autoimmune pancreatitis	Alopecia areata	Juvenile myositis (JM)	Scleroderma	Intracranial hemorrhage
Myocardial infarction	Autoimmune encephalomyelitis	Pemphigus	Eosinophilic fasciitis	Vitiligo	Castleman disease (CD)

Vertigo	Multiple sclerosis	Linear IgA disease (LAD)	Essential mixed cryoglobulinemia	Other Immune responses	Lymphopenia
Myocarditis	Transverse myelitis	Goodpastures syndrome	Pure red cell aplasia (PRCA)	Dermatomyositis	Bullous pemphigoid Giant cell Primary sclerosing
IgG4-related Hidradenitis Suppurativa myocarditis cholangitis sclerosing disease CREST syndrome (HS) (Acne Inversa) Crohns disease					
Fibrosing alveolitis	Polymyalgia rheumatica	Behcets disease	Postmyocardial infarction syndrome	Microscopic polyangiitis (MPA)	Sjögrens syndrome
Autoimmune urticarial	Pars planitis (peripheral uveitis)	Benign mucosal pemphigoid	Inclusion body myositis (IBM)	Autoimmune hepatitis	Addisons disease
Perivenous encephalomyelitis	Uveitis	Other thrombosis	Palindromic rheumatism (PR)	Juvenile arthritis	Retroperitoneal fibrosis
Paresthesia	Vogt-Koyanagi-Harada Disease	Baló disease	Chronic recurrent multifocal osteomyelitis (CRMO)	Motor neuron disease	Autoimmune angioedema
Rheumatic fever	Peripheral neuropathy	Cogans syndrome	Paroxysmal nocturnal hemoglobinuria (PNH)	Dermatitis herpetiformis	Parry Romberg syndrome
Myositis	Discoid lupus	Susacs syndrome	Temporal arteritis/Giant cell arteritis	Neutropenia	Thyroid eye disease (TED)
Lymphadenopathy	IgA Nephropathy	Congenital heart block	Giant cell arteritis (temporal arteritis)	Autoimmune dysautonomia	Sarcoidosis
Axonal & neuronal neuropathy (AMAN)	Primary Biliary Cholangitis	Hashimotos thyroiditis	Amyloidosis	Celiac disease	Scleritis
Thrombocytopenia	Endometriosis	Menieres disease	Agammaglobulinemia	Psoriatic arthritis	Ulcerative colitis (UC)
Guillain-Barre syndrome	Vasculitis	Cicatricial pemphigoid	Restless legs syndrome (RLS)/Stiff person syndrome (SPS)	Relapsing polychondritis	Cold agglutinin disease
Autoimmune myocarditis	Postpericardiotomy syndrome	Moorens ulcer	Herpes gestationis or pemphigoid gestationis (PG)	Psoriasis	Schmidt syndrome
		Raynauds phenomenon Multifocal Motor Neuropathy (MMN) or	Neonatal Lupus	Autoimmune inner ear disease (AIED)	Reflex sympathetic dystrophy

Eosinophilic esophagitis (EoE)	Parsonage-Turner syndrome	MMNCB			
Appendicitis	Hemolytic anemia		Pyoderma gangrenosum	Tolosa-Hunt syndrome (THS)	Narcolepsy

Table 4b: Data characteristics

Characteristics	Vaccination analysis	
	Vaccinated Group (N = 103,315)	Control Group (N = 103,315)
Median age (IQR) - yr		
Age group - no. (%)		
<18	(0.0)	71 (0.0)
18-39	55,363 (0.5)	56,803 (0.5)
40-49	26,111 (0.3)	25,533 (0.2)
50-59	14,612 (0.1)	14,130 (0.1)
60-69	5,762 (0.1)	5,430 (0.1)
70-79	1,302 (0.0)	1,199 (0.0)
>=80	166 (0.0)	151 (0.0)
Sex - no (%)		
Female	69,808 (67.6)	69,808 (67.6)
Male	33,507 (32.4)	33,507 (32.4)
Chronic condition count		
0	75,807 (73.4)	75,807 (73.4)
1	17,559 (17.0)	17,559 (17.0)
2	5,482 (5.3)	5,482 (5.3)
3	2,492 (2.4)	2,492 (2.4)
4+	1,977 (1.9)	1,977 (1.9)
No. of influenza vaccinations during preceding 5 yr - no(%)		
0	89,530 (86.7)	89,530 (86.7)
1	10,198 (9.9)	10,198 (9.9)
2	1,992 (1.9)	1,992 (1.9)
3	1,040 (1.0)	1,040 (1.0)
4	479 (0.5)	479 (0.5)
5	77 (0.1)	77 (0.1)

Table 4c: Conditions studied

**Condition**	**Average study population exposure (in days)**	**Average clinical twin exposure (in days)**	**Adverse events for study population (95% CI)**	**Adverse events for clinical twin population (95% CI)**	**Risk Ratio (95% CI)**
Achalasia	3,227,809	3,227,799	0.0 (0.0 to 0.0)	0.37 (0.0 to 2.0)	0.0 (0.0 to 0.0)

Condition	Average study population exposure (in days)	Average clinical twin exposure (in days)	Adverse events for study population (95% CI)	Adverse events for clinical twin population (95% CI)	Risk Ratio (95% CI)
Acute kidney injury	3,189,599	3,189,597	6.32 (2.0 to 12.0)	7.01 (2.0 to 13.0)	1.13 (0.29 to 3.85)
Addisons disease	3,232,734	3,232,734	0.0 (0.0 to 0.0)	0.0 (0.0 to 0.0)	0.0 (nan to nan)
Adult Still's disease	3,231,550	3,231,549	0.0 (0.0 to 0.0)	0.03 (0.0 to 1.0)	0.0 (0.0 to 0.0)
Agammaglobulinemia	3,227,528	3,227,527	0.0 (0.0 to 0.0)	0.04 (0.0 to 1.0)	0.0 (0.0 to 0.0)
Alopecia areata	3,187,925	3,187,813	2.8 (0.0 to 6.0)	7.61 (3.0 to 13.0)	0.42 (0.0 to 1.25)
Amyloidosis	3,232,258	3,232,258	0.0 (0.0 to 0.0)	0.0 (0.0 to 0.0)	0.0 (0.0 to 0.0)
Anemia	2,667,434	2,667,252	37.17 (26.0 to 48.0)	47.8 (35.0 to 61.0)	0.79 (0.51 to 1.16)
Ankylosing spondylitis	3,216,146	3,216,117	0.9 (0.0 to 3.0)	1.58 (0.0 to 4.0)	0.59 (0.0 to 2.0)
Anti-GBM/Anti-TBM nephritis	3,224,889	3,224,886	0.0 (0.0 to 0.0)	0.17 (0.0 to 1.0)	0.0 (0.0 to 0.0)
Antiphospholipid syndrome	3,230,575	3,230,569	0.2 (0.0 to 1.0)	0.36 (0.0 to 2.0)	0.16 (0.0 to 1.0)
Appendicitis	3,070,563	3,070,436	12.34 (6.0 to 21.0)	16.88 (9.0 to 25.0)	0.78 (0.31 to 1.5)
Arrhythmia	3,036,318	3,036,451	23.31 (14.0 to 33.0)	24.79 (16.0 to 35.0)	0.98 (0.52 to 1.71)
Arthritis or arthropathy	2,211,811	2,211,869	66.76 (51.0 to 83.0)	63.92 (50.0 to 79.52)	1.06 (0.74 to 1.49)
Autoimmune angioedema	3,231,821	3,231,821	0.0 (0.0 to 0.0)	0.0 (0.0 to 0.0)	0.0 (nan to nan)
Autoimmune dysautonomia	3,228,278	3,228,275	0.73 (0.0 to 2.0)	0.95 (0.0 to 3.0)	0.53 (0.0 to 2.0)
Autoimmune encephalomyelitis	3,224,649	3,224,625	0.0 (0.0 to 0.0)	0.71 (0.0 to 3.0)	0.0 (0.0 to 0.0)
Autoimmune hepatitis	3,230,609	3,230,605	0.0 (0.0 to 0.0)	0.13 (0.0 to 1.0)	0.0 (0.0 to 0.0)
Autoimmune inner ear disease (AIED)	3,202,773	3,202,796	4.38 (1.0 to 9.0)	2.69 (0.0 to 6.0)	1.99 (0.25 to 6.0)
Autoimmune myocarditis	3,225,825	3,225,820	0.0 (0.0 to 0.0)	0.35 (0.0 to 2.0)	0.0 (0.0 to 0.0)

Autoimmune oophoritis	3,147,456	3,147,459	7.47 (3.0 to 13.0)	7.31 (2.0 to 13.0)	1.23 (0.36 to 3.42)
Autoimmune orchitis	3,172,859	3,172,955	8.21 (3.0 to 14.0)	5.35 (1.0 to 10.52)	1.97 (0.43 to 6.0)
Autoimmune pancreatitis	3,219,995	3,219,983	0.0 (0.0 to 0.0)	0.75 (0.0 to 3.0)	0.0 (0.0 to 0.0)
Autoimmune retinopathy	3,145,920	3,145,928	10.34 (4.0 to 17.0)	10.79 (5.0 to 18.0)	1.07 (0.39 to 2.5)
Autoimmune urticarial	2,946,721	2,946,549	19.25 (11.0 to 28.52)	28.56 (19.0 to 39.0)	0.7 (0.34 to 1.12)
Axonal & neuronal neuropathy (AMAN)	3,219,677	3,219,645	0.9 (0.0 to 3.0)	2.75 (0.0 to 6.0)	0.42 (0.0 to 2.0)
Baló disease	3,232,634	3,232,634	0.0 (0.0 to 0.0)	0.0 (0.0 to 0.0)	0.0 (nan to nan)
Behcets disease	3,232,734	3,232,734	0.0 (0.0 to 0.0)	0.0 (0.0 to 0.0)	0.0 (nan to nan)
Bells palsy	3,232,734	3,232,734	0.0 (0.0 to 0.0)	0.0 (0.0 to 0.0)	0.0 (nan to nan)

**Condition**	**Average study population exposure (in days)**	**Average clinical twin exposure (in days)**	**Adverse events for study population (95% CI)**	**Adverse events for clinical twin population (95% CI)**	**Risk Ratio (95% CI)**
Benign mucosal pemphigoid	3,232,258	3,232,258	0.0 (0.0 to 0.0)	0.0 (0.0 to 0.0)	0.0 (nan to nan)
Bullous pemphigoid	3,231,658	3,231,656	0.0 (0.0 to 0.0)	0.06 (0.0 to 1.0)	0.0 (0.0 to 0.0)
CREST syndrome	3,231,835	3,231,835	0.0 (0.0 to 0.0)	0.0 (0.0 to 0.0)	0.0 (nan to nan)
Castleman disease (CD)	3,225,441	3,225,424	0.0 (0.0 to 0.0)	0.62 (0.0 to 2.0)	0.0 (0.0 to 0.0)
Celiac disease	3,225,081	3,225,068	0.0 (0.0 to 0.0)	0.61 (0.0 to 3.0)	0.0 (0.0 to 0.0)
Cerebrovascular accident	3,147,313	3,147,296	7.6 (3.0 to 13.0)	9.08 (4.0 to 15.52)	0.95 (0.31 to 2.59)
Chagas disease	3,232,180	3,232,180	0.0 (0.0 to 0.0)	0.0 (0.0 to 0.0)	0.0 (nan to nan)
Chronic inflammatory demyelinating polyneuropathy (CIDP)	3,225,249	3,225,209	0.0 (0.0 to 0.0)	1.99 (0.0 to 5.0)	0.0 (0.0 to 0.0)
Chronic recurrent multifocal osteomyelitis (CRMO)	3,232,038	3,232,036	0.0 (0.0 to 0.0)	0.12 (0.0 to 1.0)	0.0 (0.0 to 0.0)
Churg-Strauss Syndrome (CSS) or Eosinophilic Granulomatosis (EGPA)	3,232,372	3,232,370	0.0 (0.0 to 0.0)	0.08 (0.0 to 1.0)	0.0 (0.0 to 0.0)

Cicatricial pemphigoid	3,232,258	3,232,258	0.0 (0.0 to 0.0)	0.0 (0.0 to 0.0)	0.0 (nan to nan)
Cogans syndrome	3,232,734	3,232,734	0.0 (0.0 to 0.0)	0.0 (0.0 to 0.0)	0.0 (nan to nan)
Cold agglutinin disease	3,231,446	3,231,443	0.0 (0.0 to 0.0)	0.09 (0.0 to 1.0)	0.0 (0.0 to 0.0)
Congenital heart block	3,232,134	3,232,134	0.0 (0.0 to 0.0)	0.02 (0.0 to 0.0)	0.0 (0.0 to 0.0)
Coxsackie myocarditis	3,225,824	3,225,824	0.0 (0.0 to 0.0)	0.0 (0.0 to 0.0)	0.0 (nan to nan)
Crohns disease	3,232,734	3,232,734	0.0 (0.0 to 0.0)	0.0 (0.0 to 0.0)	0.0 (nan to nan)
Deep-vein thrombosis	3,105,790	3,105,901	17.26 (10.0 to 26.0)	12.11 (6.0 to 19.0)	1.56 (0.7 to 3.13)
Dermatitis herpetiformis	3,229,167	3,229,164	0.0 (0.0 to 0.0)	0.16 (0.0 to 1.0)	0.0 (0.0 to 0.0)
Dermatomyositis	3,230,704	3,230,703	0.0 (0.0 to 0.0)	0.04 (0.0 to 1.0)	0.0 (0.0 to 0.0)
Devics disease (neuromyelitis optica)	3,232,734	3,232,734	0.0 (0.0 to 0.0)	0.0 (0.0 to 0.0)	0.0 (nan to nan)
Discoid lupus	3,226,370	3,226,390	1.39 (0.0 to 4.0)	0.41 (0.0 to 2.0)	1.39 (0.0 to 4.0)
Dresslers syndrome	3,232,734	3,232,734	0.0 (0.0 to 0.0)	0.0 (0.0 to 0.0)	0.0 (nan to nan)
Endometriosis	2,905,162	2,905,088	22.67 (14.0 to 33.0)	22.19 (14.0 to 31.0)	1.07 (0.59 to 1.78)
Eosinophilic esophagitis (EoE)	3,135,425	3,135,350	3.39 (1.0 to 7.0)	6.04 (2.0 to 11.0)	0.68 (0.07 to 2.0)
Eosinophilic fasciitis	3,232,380	3,232,379	0.0 (0.0 to 0.0)	0.05 (0.0 to 1.0)	0.0 (0.0 to 0.0)

**Condition**	**Average study population exposure (in days)**	**Average clinical twin exposure (in days)**	**Adverse events for study population (95% CI)**	**Adverse events for clinical twin population (95% CI)**	**Risk Ratio (95% CI)**
Erythema nodosum	3,224,679	3,224,701	0.89 (0.0 to 3.0)	0.44 (0.0 to 2.0)	0.84 (0.0 to 3.0)
Essential mixed cryoglobulinemia	3,232,565	3,232,565	0.0 (0.0 to 0.0)	0.01 (0.0 to 0.0)	0.0 (0.0 to 0.0)
Evans syndrome	3,226,511	3,226,534	1.63 (0.0 to 4.0)	0.11 (0.0 to 1.0)	1.62 (0.0 to 3.88)
Fibromyalgia	3,184,600	3,184,625	9.93 (5.0 to 16.0)	10.17 (4.48 to 17.0)	1.11 (0.42 to 2.6)

Fibrosing alveolitis	3,222,706	3,222,690	0.0 (0.0 to 0.0)	0.64 (0.0 to 2.0)	0.0 (0.0 to 0.0)
Giant cell arteritis (temporal arteritis)	3,231,663	3,231,662	0.0 (0.0 to 0.0)	0.12 (0.0 to 1.0)	0.0 (0.0 to 0.0)
Giant cell myocarditis	3,221,587	3,221,573	0.0 (0.0 to 0.0)	1.01 (0.0 to 3.0)	0.0 (0.0 to 0.0)
Glomerulonephritis	3,009,371	3,009,279	14.5 (8.0 to 22.0)	16.95 (9.0 to 25.0)	0.91 (0.41 to 1.71)
Goodpastures syndrome	3,232,734	3,232,734	0.0 (0.0 to 0.0)	0.0 (0.0 to 0.0)	0.0 (nan to nan)
Granulomatosis with Polyangiitis	3,232,700	3,232,700	0.0 (0.0 to 0.0)	0.0 (0.0 to 0.0)	0.0 (nan to nan)
Graves disease	3,232,734	3,232,734	0.0 (0.0 to 0.0)	0.0 (0.0 to 0.0)	0.0 (nan to nan)
Guillain-Barre syndrome	3,226,331	3,226,334	0.92 (0.0 to 3.0)	1.05 (0.0 to 3.0)	0.7 (0.0 to 3.0)
Hashimotos thyroiditis	3,232,734	3,232,734	0.0 (0.0 to 0.0)	0.0 (0.0 to 0.0)	0.0 (nan to nan)
Hemolytic anemia	3,231,446	3,231,443	0.0 (0.0 to 0.0)	0.09 (0.0 to 1.0)	0.0 (0.0 to 0.0)
Henoch-Schonlein purpura (HSP)	3,224,358	3,224,337	0.0 (0.0 to 0.0)	0.86 (0.0 to 3.0)	0.0 (0.0 to 0.0)
Herpes gestationis or pemphigoid gestationis (PG)	3,230,789	3,230,784	0.0 (0.0 to 0.0)	0.18 (0.0 to 1.0)	0.0 (0.0 to 0.0)
Herpes simplex infection	2,971,196	2,971,120	17.93 (10.48 to 26.0)	24.76 (16.0 to 36.0)	0.76 (0.36 to 1.29)
Herpes zoster infection	3,076,109	3,076,207	20.08 (11.48 to 30.0)	21.04 (13.0 to 30.0)	0.99 (0.5 to 1.64)
Hidradenitis Suppurativa (HS) (Acne Inversa)	3,208,853	3,208,808	2.15 (0.0 to 5.0)	3.38 (0.0 to 7.0)	0.84 (0.0 to 3.0)
Hypersensitivity pneumonitis	3,214,211	3,214,192	0.0 (0.0 to 0.0)	1.0 (0.0 to 3.0)	0.0 (0.0 to 0.0)
Hyperthyroidism	3,154,251	3,154,146	2.18 (0.0 to 5.52)	5.81 (1.0 to 11.0)	0.5 (0.0 to 2.0)
Hypogammalglobulinemia	3,232,248	3,232,248	0.0 (0.0 to 0.0)	0.0 (0.0 to 0.0)	0.0 (nan to nan)
IgA Nephropathy	3,199,927	3,199,886	0.7 (0.0 to 3.0)	2.24 (0.0 to 6.0)	0.38 (0.0 to 2.0)
IgG4-related sclerosing disease	3,232,137	3,232,136	0.0 (0.0 to 0.0)	0.04 (0.0 to 1.0)	0.0 (0.0 to 0.0)
Immune thrombocytopenic purpura (ITP)	3,227,052	3,227,075	1.63 (0.0 to 4.0)	0.11 (0.0 to 1.0)	1.62 (0.0 to 3.88)
Inclusion body myositis (IBM)	3,231,478	3,231,476	0.0 (0.0 to 0.0)	0.08 (0.0 to 1.0)	0.0 (0.0 to 0.0)

Condition	Average study population exposure (in days)	Average clinical twin exposure (in days)	Adverse events for study population (95% CI)	Adverse events for clinical twin population (95% CI)	Risk Ratio (95% CI)
Interstitial cystitis (IC)	3,207,419	3,207,450	3.95 (0.0 to 8.0)	2.18 (0.0 to 5.0)	2.24 (0.0 to 7.0)
Intracranial hemorrhage	3,201,232	3,201,238	2.25 (0.0 to 6.0)	2.15 (0.0 to 5.0)	1.24 (0.0 to 4.0)
Juvenile arthritis	3,226,829	3,226,826	0.0 (0.0 to 0.0)	0.18 (0.0 to 1.52)	0.0 (0.0 to 0.0)
Juvenile diabetes (Type 1 diabetes)	3,103,501	3,103,376	5.09 (1.0 to 9.0)	9.33 (4.0 to 15.0)	0.63 (0.13 to 1.75)
Juvenile myositis (JM)	3,232,071	3,232,071	0.0 (0.0 to 0.0)	0.0 (0.0 to 0.0)	0.0 (0.0 to 0.0)
Kawasaki disease	3,232,398	3,232,390	0.0 (0.0 to 0.0)	1.1 (0.0 to 3.0)	0.0 (0.0 to 0.0)
Lambert-Eaton syndrome	3,232,609	3,232,609	0.0 (0.0 to 0.0)	0.0 (0.0 to 0.0)	0.0 (nan to nan)
Leukocytoclastic vasculitis	3,209,970	3,209,957	0.52 (0.0 to 2.0)	1.52 (0.0 to 5.0)	0.33 (0.0 to 1.95)
Lichen planus	3,213,599	3,213,626	1.85 (0.0 to 5.0)	1.31 (0.0 to 4.0)	1.39 (0.0 to 4.0)
Lichen sclerosus	3,225,397	3,225,369	0.0 (0.0 to 0.0)	0.96 (0.0 to 3.0)	0.0 (0.0 to 0.0)
Ligneous conjunctivitis	2,332,156	2,332,213	53.94 (40.0 to 69.0)	48.6 (36.0 to 62.52)	1.13 (0.76 to 1.65)
Linear IgA disease (LAD)	3,227,807	3,227,825	0.84 (0.0 to 3.0)	0.32 (0.0 to 2.0)	0.75 (0.0 to 2.0)
Lupus	3,194,102	3,194,050	4.22 (1.0 to 8.0)	5.7 (1.0 to 11.0)	0.96 (0.1 to 3.5)
Lyme disease chronic	3,232,010	3,232,031	0.9 (0.0 to 3.0)	0.2 (0.0 to 1.0)	0.87 (0.0 to 3.0)
Lymphadenopathy	2,884,700	2,884,641	19.04 (11.0 to 27.52)	19.81 (12.0 to 29.0)	1.01 (0.51 to 1.76)
Lymphopenia	3,225,715	3,225,712	0.76 (0.0 to 3.0)	0.39 (0.0 to 2.0)	0.65 (0.0 to 3.0)
Menieres disease	3,232,734	3,232,734	0.0 (0.0 to 0.0)	0.0 (0.0 to 0.0)	0.0 (nan to nan)
Microscopic polyangiitis (MPA)	3,228,638	3,228,667	1.05 (0.0 to 3.0)	0.25 (0.0 to 2.0)	0.95 (0.0 to 3.0)
Mixed connective tissue disease (MCTD)	3,229,197	3,229,219	0.94 (0.0 to 3.0)	0.44 (0.0 to 2.0)	0.86 (0.0 to 3.0)
Moorens ulcer	3,232,734	3,232,734	0.0 (0.0 to 0.0)	0.0 (0.0 to 0.0)	0.0 (nan to nan)

Motor neuron disease	3,229,370	3,229,368	0.0 (0.0 to 0.0)	0.11 (0.0 to 1.0)	0.0 (0.0 to 0.0)
Mucha-Habermann disease	3,232,097	3,232,097	0.0 (0.0 to 0.0)	0.02 (0.0 to 0.0)	0.0 (0.0 to 0.0)
Multifocal Motor Neuropathy (MMN) or MMNCB	3,232,351	3,232,351	0.0 (0.0 to 0.0)	0.0 (0.0 to 0.0)	0.0 (nan to nan)
Multiple sclerosis	3,207,647	3,207,615	0.0 (0.0 to 0.0)	1.77 (0.0 to 5.0)	0.0 (0.0 to 0.0)
Myasthenia gravis	3,227,268	3,227,263	0.0 (0.0 to 0.0)	0.37 (0.0 to 2.0)	0.0 (0.0 to 0.0)
Myelin Oligodendrocyte Glycoprotein Antibody Disorder	3,231,805	3,231,795	0.0 (0.0 to 0.0)	0.42 (0.0 to 2.0)	0.0 (0.0 to 0.0)

**Condition**	**Average study population exposure (in days)**	**Average clinical twin exposure (in days)**	**Adverse events for study population (95% CI)**	**Adverse events for clinical twin population (95% CI)**	**Risk Ratio (95% CI)**
Myocardial infarction	3,192,589	3,192,671	11.59 (6.0 to 19.0)	9.5 (4.0 to 16.0)	1.4 (0.5 to 3.13)
Myocarditis	3,214,286	3,214,271	0.0 (0.0 to 0.0)	1.02 (0.0 to 3.0)	0.0 (0.0 to 0.0)
Myositis	3,103,371	3,103,553	19.04 (12.0 to 27.52)	11.34 (5.0 to 18.0)	1.87 (0.84 to 4.0)
Narcolepsy	3,228,216	3,228,210	0.0 (0.0 to 0.0)	0.25 (0.0 to 2.0)	0.0 (0.0 to 0.0)
Neonatal Lupus	3,225,520	3,225,515	0.0 (0.0 to 0.0)	0.37 (0.0 to 2.0)	0.0 (0.0 to 0.0)
Neuromyelitis optica	3,231,805	3,231,795	0.0 (0.0 to 0.0)	0.42 (0.0 to 2.0)	0.0 (0.0 to 0.0)
Neutropenia	3,224,034	3,224,029	0.0 (0.0 to 0.0)	0.37 (0.0 to 2.0)	0.0 (0.0 to 0.0)
Ocular cicatricial pemphigoid	3,232,154	3,232,148	0.0 (0.0 to 0.0)	0.17 (0.0 to 1.0)	0.0 (0.0 to 0.0)
Optic neuritis	3,218,592	3,218,592	2.0 (0.0 to 5.0)	1.2 (0.0 to 4.0)	1.38 (0.0 to 4.0)
Other Immune responses	3,231,272	3,231,639	11.23 (5.0 to 18.0)	0.86 (0.0 to 3.0)	8.96 (3.0 to 18.0)
Other thrombosis	3,232,734	3,232,734	0.0 (0.0 to 0.0)	0.0 (0.0 to 0.0)	0.0 (nan to nan)
POEMS syndrome	3,231,495	3,231,493	0.0 (0.0 to 0.0)	0.08 (0.0 to 1.0)	0.0 (0.0 to 0.0)
Palindromic rheumatism (PR)	3,229,993	3,229,992	0.0 (0.0 to 0.0)	0.08 (0.0 to 1.0)	0.0 (0.0 to 0.0)

Paraneoplastic cerebellar degeneration (PCD)	3,231,739	3,231,739	0.0 (0.0 to 0.0)	0.0 (0.0 to 0.0)	0.0 (nan to nan)
Paresthesia	3,189,133	3,189,283	7.4 (2.48 to 13.0)	2.59 (0.0 to 6.0)	3.48 (0.6 to 10.0)
Paroxysmal nocturnal hemoglobinuria (PNH)	3,232,627	3,232,627	0.0 (0.0 to 0.0)	0.01 (0.0 to 0.0)	0.0 (0.0 to 0.0)
Parry Romberg syndrome	3,230,999	3,230,997	0.0 (0.0 to 0.0)	0.07 (0.0 to 1.0)	0.0 (0.0 to 0.0)
Pars planitis (peripheral uveitis)	3,192,728	3,192,778	4.86 (1.0 to 10.0)	3.18 (0.0 to 7.0)	1.99 (0.25 to 7.0)
Parsonage-Turner syndrome	3,231,637	3,231,637	0.0 (0.0 to 0.0)	0.0 (0.0 to 0.0)	0.0 (nan to nan)
Pemphigus	3,229,298	3,229,296	0.0 (0.0 to 0.0)	0.17 (0.0 to 1.0)	0.0 (0.0 to 0.0)
Pericarditis	3,213,907	3,213,906	1.62 (0.0 to 4.52)	1.23 (0.0 to 4.0)	1.15 (0.0 to 4.0)
Peripheral neuropathy	3,229,219	3,229,234	0.85 (0.0 to 3.0)	0.23 (0.0 to 1.0)	0.81 (0.0 to 3.0)
Perivenous encephalomyelitis	3,188,417	3,188,391	1.98 (0.0 to 5.0)	3.0 (0.0 to 7.0)	0.88 (0.0 to 3.51)
Pernicious anemia (PA)	3,223,758	3,223,755	0.0 (0.0 to 0.0)	0.18 (0.0 to 1.0)	0.0 (0.0 to 0.0)
Polyarteritis nodosa	3,229,454	3,229,485	1.05 (0.0 to 3.0)	0.16 (0.0 to 1.0)	0.99 (0.0 to 3.0)
Polyglandular syndromes type I, II, III	3,232,359	3,232,359	0.0 (0.0 to 0.0)	0.0 (0.0 to 0.0)	0.0 (nan to nan)

**Condition**	**Average study population exposure (in days)**	**Average clinical twin exposure (in days)**	**Adverse events for study population (95% CI)**	**Adverse events for clinical twin population (95% CI)**	**Risk Ratio (95% CI)**
Polymyalgia rheumatic	3,214,910	3,214,972	3.04 (0.0 to 7.0)	1.23 (0.0 to 4.0)	2.27 (0.0 to 6.0)
Polymyositis	3,227,556	3,227,610	2.91 (0.0 to 6.52)	0.62 (0.0 to 3.0)	2.55 (0.0 to 6.0)
Postmyocardial infarction syndrome	3,232,396	3,232,393	0.0 (0.0 to 0.0)	0.09 (0.0 to 1.0)	0.0 (0.0 to 0.0)
Postpericardiotomy syndrome	3,232,616	3,232,607	0.0 (0.0 to 0.0)	0.45 (0.0 to 2.0)	0.0 (0.0 to 0.0)
Primary Biliary Cholangitis	3,224,739	3,224,734	2.48 (0.0 to 6.0)	1.46 (0.0 to 4.0)	1.62 (0.0 to 5.0)
Primary sclerosing cholangitis	3,224,394	3,224,388	2.48 (0.0 to 6.0)	1.49 (0.0 to 4.0)	1.61 (0.0 to 5.0)

Progesterone dermatitis	2,340,304	2,340,372	68.9 (54.0 to 85.52)	68.57 (51.0 to 86.0)	1.02 (0.72 to 1.41)
Progressive systemic sclerosis	3,228,477	3,228,476	0.0 (0.0 to 0.0)	0.07 (0.0 to 1.0)	0.0 (0.0 to 0.0)
Psoriasis	3,150,517	3,150,499	6.34 (2.0 to 12.0)	7.87 (3.0 to 13.0)	0.96 (0.23 to 2.64)
Psoriatic arthritis	3,150,025	3,150,007	6.34 (2.0 to 12.0)	7.87 (3.0 to 13.0)	0.96 (0.23 to 2.64)
Pulmonary embolism	3,109,556	3,109,669	12.86 (7.0 to 21.0)	10.49 (5.0 to 18.0)	1.37 (0.55 to 3.11)
Pure red cell aplasia (PRCA)	3,231,132	3,231,132	0.0 (0.0 to 0.0)	0.01 (0.0 to 0.0)	0.0 (0.0 to 0.0)
Pyoderma gangrenosum	3,231,641	3,231,641	0.0 (0.0 to 0.0)	0.16 (0.0 to 1.0)	0.0 (0.0 to 0.0)
Raynauds phenomenon	3,232,734	3,232,734	0.0 (0.0 to 0.0)	0.0 (0.0 to 0.0)	0.0 (nan to nan)
Reactive Arthritis	3,222,973	3,223,023	3.21 (0.0 to 7.0)	1.03 (0.0 to 3.0)	2.42 (0.0 to 6.0)
Reflex sympathetic dystrophy	3,231,171	3,231,183	1.35 (0.0 to 4.0)	0.14 (0.0 to 1.0)	1.23 (0.0 to 3.0)
Relapsing polychondritis	3,230,175	3,230,180	0.53 (0.0 to 2.0)	0.37 (0.0 to 2.0)	0.5 (0.0 to 2.0)
Restless legs syndrome (RLS)/Stiff person syndrome (SPS)	3,222,348	3,222,312	0.0 (0.0 to 0.0)	2.0 (0.0 to 5.0)	0.0 (0.0 to 0.0)
Retroperitoneal fibrosis	3,229,267	3,229,264	0.0 (0.0 to 0.0)	0.19 (0.0 to 1.0)	0.0 (0.0 to 0.0)
Rheumatic fever	3,208,940	3,208,952	1.57 (0.0 to 4.0)	1.67 (0.0 to 4.0)	0.95 (0.0 to 3.45)
Rheumatoid arthritis	3,085,558	3,085,571	11.77 (5.0 to 19.0)	14.25 (7.0 to 22.0)	0.9 (0.34 to 1.86)
Sarcoidosis	3,220,129	3,220,143	1.0 (0.0 to 3.0)	0.61 (0.0 to 2.0)	0.82 (0.0 to 3.0)
Schmidt syndrome	3,232,359	3,232,359	0.0 (0.0 to 0.0)	0.0 (0.0 to 0.0)	0.0 (nan to nan)
Scleritis	3,214,650	3,214,630	0.99 (0.0 to 3.0)	1.75 (0.0 to 5.0)	0.61 (0.0 to 3.0)
Scleroderma	3,228,608	3,228,603	0.0 (0.0 to 0.0)	0.17 (0.0 to 1.0)	0.0 (0.0 to 0.0)
**Condition**	**Average study population exposure (in days)**	**Average clinical twin exposure (in days)**	**Adverse events for study population (95% CI)**	**Adverse events for clinical twin population (95% CI)**	**Risk Ratio (95% CI)**
Seizure	2,984,509	2,984,396	19.37 (12.0 to 28.52)	25.25 (16.0 to 35.0)	0.8 (0.44 to 1.37)

Sjögrens syndrome	3,232,734	3,232,734	0.0 (0.0 to 0.0)	0.0 (0.0 to 0.0)	0.0 (nan to nan)
Sperm & testicular autoimmunity	3,213,695	3,213,691	0.96 (0.0 to 3.0)	1.41 (0.0 to 4.0)	0.67 (0.0 to 3.0)
Subacute bacterial endocarditis (SBE)	3,228,351	3,228,343	0.0 (0.0 to 0.0)	0.37 (0.0 to 2.0)	0.0 (0.0 to 0.0)
Susacs syndrome	3,232,734	3,232,734	0.0 (0.0 to 0.0)	0.0 (0.0 to 0.0)	0.0 (nan to nan)
Sympathetic ophthalmia (SO)	3,231,701	3,231,733	1.02 (0.0 to 3.0)	0.12 (0.0 to 1.0)	1.06 (0.0 to 3.0)
Syncope	2,961,279	2,961,344	23.19 (14.0 to 33.0)	22.75 (14.0 to 32.52)	1.07 (0.57 to 1.88)
Takayasus arteritis	3,232,734	3,232,734	0.0 (0.0 to 0.0)	0.0 (0.0 to 0.0)	0.0 (nan to nan)
Temporal arteritis/Giant cell arteritis	3,231,857	3,231,855	0.0 (0.0 to 0.0)	0.12 (0.0 to 1.0)	0.0 (0.0 to 0.0)
Thrombocytopenia	3,200,216	3,200,232	2.56 (0.0 to 6.0)	1.88 (0.0 to 5.0)	1.52 (0.0 to 5.58)
Thrombocytopenic purpura (TTP)	3,227,052	3,227,075	1.63 (0.0 to 4.0)	0.11 (0.0 to 1.0)	1.62 (0.0 to 3.88)
Thyroid eye disease (TED)	3,204,746	3,204,729	1.67 (0.0 to 5.0)	2.6 (0.0 to 6.0)	0.82 (0.0 to 3.0)
Tolosa-Hunt syndrome (THS)	3,227,277	3,227,266	0.0 (0.0 to 0.0)	0.55 (0.0 to 2.0)	0.0 (0.0 to 0.0)
Transverse myelitis	3,229,760	3,229,797	1.76 (0.0 to 5.0)	0.26 (0.0 to 1.52)	1.68 (0.0 to 5.0)
Type 1 diabetes	3,103,501	3,103,376	5.09 (1.0 to 9.0)	9.33 (4.0 to 15.0)	0.63 (0.13 to 1.75)
Ulcerative colitis (UC)	3,157,352	3,157,336	4.61 (1.0 to 9.0)	4.73 (1.0 to 10.0)	1.31 (0.23 to 5.0)
Undifferentiated connective tissue disease (UCTD)	3,229,197	3,229,219	0.94 (0.0 to 3.0)	0.44 (0.0 to 2.0)	0.86 (0.0 to 3.0)
Uveitis	3,193,094	3,193,144	4.86 (1.0 to 10.0)	3.18 (0.0 to 7.0)	1.99 (0.25 to 7.0)
Vasculitis	3,208,063	3,208,046	0.52 (0.0 to 2.0)	1.65 (0.0 to 5.0)	0.32 (0.0 to 1.0)
Vertigo	2,864,193	2,864,604	51.75 (39.0 to 66.52)	42.37 (30.0 to 55.52)	1.25 (0.83 to 1.91)
Vitiligo	3,218,418	3,218,424	2.05 (0.0 to 5.0)	1.07 (0.0 to 3.0)	1.52 (0.0 to 4.58)
Vogt-Koyanagi-Harada Disease	3,193,094	3,193,144	4.86 (1.0 to 10.0)	3.18 (0.0 to 7.0)	1.99 (0.25 to 7.0)
